# Metabolic dysfunction-associated steatotic liver disease and increased risk of atrial fibrillation in the elderly: A longitudinal cohort study

**DOI:** 10.1016/j.ijcha.2025.101676

**Published:** 2025-04-08

**Authors:** Yehua Tang, Jianling Fan, Xingyun Hou, Honghong Wu, Jiaqi Zhang, Jia Wu, Yifan Wang, Zhiyu Zhang, Bin Lu, Jiaoyang Zheng

**Affiliations:** a Department of Cardiology, Second Affiliated Hospital of Naval Medical University, Shanghai 200003, China; b Health Management Centre, Second Affiliated Hospital of Naval Medical University, Shanghai 200003, China; c Department of Pharmacy, Second Affiliated Hospital of Naval Medical University, Shanghai 200003, China; d Department of Biochemical Pharmacy, School of Pharmacy, Naval Medical University, Shanghai 200433, China

**Keywords:** Cohort analysis, Metabolic dysfunction-associated steatotic liver disease, Atrial fibrillation, Cardiometabolic risk factors

## Abstract

**Background:**

Emerging evidence suggests a link between metabolic dysfunction-associated steatotic liver disease (MASLD) and cardiac arrhythmia. This study aims to investigate the potential relationship between MASLD and atrial fibrillation (AF).

**Methods:**

This retrospective cohort study included 8511 participants (age > 65 years) without a history of cardiovascular diseases, cancer, or severe kidney dysfunction. MASLD was diagnosed using hepatic ultrasound in the presence of at least one cardiometabolic risk factor. Poisson regression models were employed to estimate the relative risk (RR) of AF, adjusting for potential confounders.

**Results:**

Participants were categorized into MASLD (n = 3,926) and non-MASLD (n = 4,585) groups. During a mean follow-up period of 3.65 ± 1.20 years, 307 participants with MASLD developed AF, however, the number in the non-MASLD group was 144 (incidence rate 7.82 % vs. 3.14 %). After adjusting for multiple cardiovascular risk factors, MASLD was associated with increased risk of AF (RR = 1.55, 95 %, confidence interval (CI): 1.12–2.13). Positive correlations were observed between age, body mass index (BMI), systolic and diastolic blood pressure, low-density lipoprotein levels, and AF risk. Subgroup analysis revealed a stronger association between MASLD and AF in participants with BMI < 24 kg/m^2^ (P < 0.01).

**Conclusion:**

This study highlights a significant association between MASLD and an increased risk of developing AF. The elevated risk in patients with MASLD may involve mechanisms extending beyond traditional cardiometabolic factors, particularly in individuals with lower BMI. Further experimental research is warranted to elucidate the underlying pathways linking MASLD and AF.

## 1 Introduction

Metabolic dysfunction-associated steatotic liver disease (MASLD), previously known as non-alcoholic fatty liver disease (NAFLD), is the most prevalent liver and metabolic disorder globally [1], [2], affecting over one-third of the adult population worldwide [3]. In 2020, the International Liver Disease Consensus Group recommended renaming NAFLD to metabolism-associated fatty liver disease (MAFLD) to underscore the role of metabolic risk factors, such as type 2 diabetes and obesity, in the disease's development. By 2023, the major European and American hepatology societies adopted the term MASLD, further broadening the diagnostic criteria for steatotic liver disease [4]. Beyond its liver-specific consequences, MASLD is a significant risk factor for various systemic conditions, including cardiovascular disease, chronic kidney disease, and extrahepatic malignancies [5], [6], [7], and it imposes a considerable economic burden on healthcare systems [3], [8].

Atrial fibrillation (AF), the most common cardiac arrhythmia, continues to rise in prevalence [9], [10]. Severe AF can lead to thrombosis, stroke, cardiac remodelling, heart failure, and sudden cardiac arrest [11], [12], [13]. As such, preventing and managing AF has become a pressing public health concern [14], [15]. Emerging evidence suggests a potential association between MASLD and cardiac arrhythmias, including AF [16], [17], [18]. However, the current research is limited and presents conflicting findings. To address this gap, we conducted a retrospective cohort study to investigate the incidence of AF in individuals with and without MASLD at baseline.

## 2 Methods

### 2.1 Study design and population

**Fig. 1:**
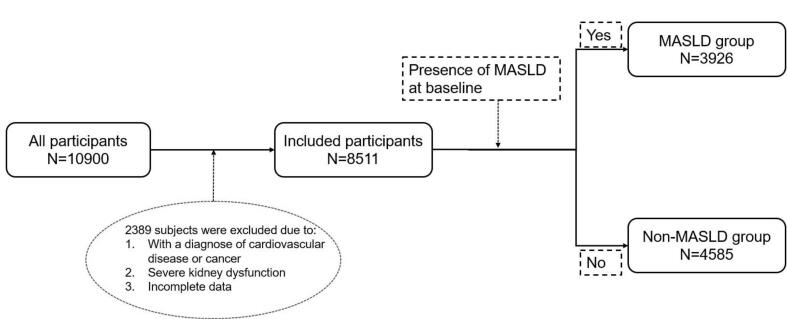
Flow chart of study participants.

This study utilized data from the Health Management Centre of the Second Affiliated Hospital of Naval Medical University, collected between January 2018 and June 2023, as previously described [19]. Participants aged over 65 years were included in the analysis. Additional inclusion criteria required complete records of demographic information, clinical measurements, and laboratory test results. Individuals with a history of cardiovascular diseases, cancer, or severe kidney dysfunction were excluded from the study (see flow chart in Fig. 1). All participants provided informed consent during their annual check-ups. The study protocol was approved by the Institutional Review Board of the Second Affiliated Hospital of Naval Medical University (2023SL052).

### 2.2 Measurements and data collection

Data were collected during routine health examinations. Participants fasted for at least 12 h before their appointments to ensure accurate blood test results. Height and weight were measured using standardized equipment, with participants wearing light clothing and no shoes. Height was recorded to the nearest 0.1 cm and weight to the nearest 0.1 kg. Body mass index (BMI) was calculated as weight (kg) divided by height squared (m^2^). Blood pressure was measured using an automated sphygmomanometer after participants had been seated and rested for a minimum of 5 min. Two readings were taken at a 3-minute interval, with the average used in the analysis. Medical histories, including diabetes, hypertension, and dyslipidaemia, were obtained via a standardized questionnaire. To reduce recall bias, diagnoses were validated through chart reviews for a subset of 1090 participants, yielding a diagnostic agreement rate of 92.4 %, with 83 participants reporting incorrect diagnoses.

Laboratory analyses included fasting plasma glucose, lipid profiles, haemoglobin A1c (HbA1c), and assessments of renal and liver function. Fasting plasma glucose and serum lipid profiles containing total cholesterol (TC), low density lipoprotein cholesterol (LDL-C), high density lipoprotein cholesterol −cholesterol (HDL-C) and triglycerides (TG), along with thyroid-stimulating hormone (TSH) and high-sensitivity C-reactive protein (hs-CRP), were measured using standard enzymatic methods. HbA1c levels were determined via high-performance liquid chromatography (HPLC). All laboratory procedures followed strict clinical protocols to ensure consistency and reliability of the data.

### 2.3 Hepatic ultrasonography

All participants underwent hepatic ultrasound examinations conducted by a professional sonographer blinded to their clinical characteristics. The ultrasounds were performed using a 5.0 MHz transducer (EPIQ 7, Philips Healthcare, Cambridge, MA, USA). Images of both the liver and kidney regions were captured and subsequently analysed. To standardize the ultrasound liver/kidney echo intensity ratio and the liver echo intensity attenuation rate, NIH-certified imaging software (ImageJ 1.41o, NIH, Bethesda, MD) was utilized. Hepatic steatosis was defined based on liver fat content, calculated using the equation: Liver Fat Content (%) = 61.519 × (ultrasound hepatic/renal ratio) + 167.701 × (hepatic echo-intensity attenuation rate) − 26.736 [20]. Hepatic steatosis was diagnosed when the calculated liver fat content was ≥ 10.0 %.

### 2.4 Definitions and outcomes

MASLD was defined according to the most recent Delphi consensus statements. Participants were diagnosed with MASLD if hepatic steatosis was detected by ultrasound and at least one of the following five cardiometabolic risk factors was present: (1) BMI ≥ 23 kg/m^2^ or waist circumference > 94 cm in men or > 80 cm in women. (2) Fasting serum glucose ≥ 5.6 mmol/L, 2-hour plasma glucose ≥ 7.8 mmol/L, HbA1c ≥ 5.7 %, or a diagnosis or treatment for type 2 diabetes. (3) Blood pressure ≥ 130/85 mmHg or treatment with antihypertensive medications. (4) TG ≥ 1.70 mmol/L or treatment with lipid-lowering medications. (5) HDL-C ≤ 1.0 mmol/L in men or ≤ 1.3 mmol/L in women or treatment with lipid-lowering medications. The primary outcome was the incidence of AF. AF was identified based on self-reported history validated using ICD-10-CM codes (I48.x) or through electrocardiogram (EKG) findings. EKG criteria for AF included the absence of discrete P waves and irregularly irregular R-R intervals on a 12-lead EKG or 24-hour Holter monitoring. Incident AF events included both paroxysmal and persistent AF.

### 2.5 Statistical analysis

Between-group comparisons were conducted using the chi-square test for categorical variables and independent *t*-tests for continuous variables. We used propensity score matching to balance the baseline characteristics. Poisson regression models were used to estimate RRs with 95 % CIs. The analysis included three models: Model 1 (Crude Model): MASLD as the sole predictor. Model 2: Adjusted for age and sex. Model 3: Further adjusted for BMI, systolic blood pressure (SBP), LDL-C, HDL-C, TG, hs-CRP, gamma-glutamyl transferase (GGT), estimated glomerular filtration rate (eGFR) and all the medications. The significance of covariates was verified using fully adjusted Poisson regression models (see Table 2). Covariate selection was based on their statistical relevance to AF risk. All statistical analyses were performed using R software (version 4.3.3, R Foundation for Statistical Computing, Vienna, Austria). A two-tailed P value < 0.05 was considered statistically significant.

**Table 1:** Baseline characteristics of participants based on presence of MASLD at baseline.

**Characteristic**	**Non-MASLD (n = 4585)**	**MASLD (n = 3926)**
Age(years)	72.78 ± 6.48	73.17 ± 6.61
BMI (kg/m^2^)	24.15 ± 2.45	24.24 ± 4.32
Male (%)	2762 (60.24 %)	2391 (60.90 %)
Female (%)	1823(39.76 %)	1535(39.10 %)
Systolic blood pressure (mmHg)	132.88 ± 13.67	133.43 ± 14.10
Diastolic blood pressure (mmHg)	81.10 ± 7.98	80.89 ± 8.51
Albumin (g/L)	43.41 ± 2.05	43.41 ± 2.08
Total bilirubin (umol/L)	12.50 ± 2.94	12.51 ± 2.94
Gamma-glutamyl transferase (U/L)	31.95 ± 19.60	30.63 ± 25.53
Uric acid (umol/L)	351.51 ± 84.04	351.20 ± 81.56
Fasting plasma glucose (mmol/L)	5.98 ± 1.46	5.97 ± 1.46
Hemoglobin A1c (%)	6.21 ± 0.59	6.20 ± 0.58
LDL-cholesterol (mmol/L)	3.21 ± 0.57	3.21 ± 0.61
Triglycerides (mmol/L)	1.58 ± 0.63	1.57 ± 0.70
HDL-cholesterol (mmol/L)	1.36 ± 0.22	1.36 ± 0.23
Total cholesterol (mmol/L)	5.07 ± 0.97	5.02 ± 0.97
Anti-diabetes medications (%)	1000 (21.81 %)	2020 (51.45 %) *
Lipid-lowering medications (%)	0 (0.00 %)	2957 (75.32 %) *
Anti-hypertension medications (%)	2843 (62.01 %)	3768 (95.98 %) *

Data are presented as mean ± standard deviation (SD). *P < 0.05.

**Table 2:** Covariates in the Poisson regression model.

	Coefficient	P
Age	0.051	0.015
Body mass index	0.077	<0.001
Systolic Blood Pressure (SBP)	0.015	<0.001
Diastolic Blood Pressure (DBP)	0.025	<0.001
LDL-cholesterol (LDL-c)	0.472	<0.001
Triglycerides (TG)	−1.770	<0.001
Anti-diabetes medications	−1.641	<0.001
Lipid-lowering medications	−0.997	<0.001
Anti-hypertension medications	1.476	<0.001

## 3 Results

This study included 10,900 adult participants. Of these, 2,389 were excluded due to a history of cardiovascular disease, cancer, severe renal insufficiency, or incomplete data, leaving a final sample of 8,511 participants for analysis. Among them, 3,926 participants (2,391 males [60.90 %] and 1,535 females [39.10 %]) met the diagnostic criteria for MASLD at baseline, while 4,585 participants (2,762 males [60.24 %] and 1,823 females [39.76 %]) did not. The flowchart detailing participant screening is presented in Fig. 1, and the descriptive characteristics of participants with and without MASLD are summarized in Table 1. The average age of participants in the MASLD group was 73.17 ± 6.61 years, compared to 72.78 ± 6.48 years in the non-MASLD group. In the non-MASLD group, 2762 (60.24 %) were male, and 1823 (39.76 %) were female. In the MASLD group, 2391 (60.90 %) were male and1535 (39.10 %) were female participants. Body mass index (BMI) was slightly higher in the MASLD group (24.24 ± 4.32 kg/m^2^) than in the non-MASLD group (24.15 ± 2.45 kg/m^2^), but the sex distribution was comparable between groups. Regarding blood pressure and biochemical parameters, including albumin, total bilirubin, uric acid, fasting plasma glucose (FPG), HbA1c, LDL-C, TG, HDL-C, and TC, no statistically significant differences were observed between the MASLD and non-MASLD groups (Table 1). However, medication usage differed markedly. A significantly higher proportion of participants in the MASLD group reported taking anti-diabetes medications (51.45 %) compared to the non-MASLD group (21.81 %). Lipid-lowering medications were used by 75.32 % of the MASLD group, while none of the non-MASLD participants reported using these treatments. Anti-hypertensive medication usage was also higher in the MASLD group (95.98 %) than in the non-MASLD group (62.01 %).

The covariates used in the Poisson regression model are detailed in Table 2. Significant positive correlations were observed between age, BMI, systolic and diastolic blood pressure, LDL-C levels, and the risk of AF in the fully adjusted model. Specifically, the risk of AF increased by 5.1 % (P = 0.015) for each 1-year increase in age and by 7.7 % (P < 0.01) for every 1-unit increase in BMI. Compared to systolic blood pressure, diastolic blood pressure had a greater impact on AF risk, with a 2.5 % (P < 0.01) increase in risk for every 1 mmHg rise. Additionally, the risk of AF increased by 47.2 % (P < 0.01) for every 1 mmol/L increase in LDL-C levels. Conversely, a significant negative correlation was observed between serum triglyceride levels and AF risk.

Over a median follow-up period of 3.65 ± 1.20 years, 307 participants in the MASLD group (7.82 %) developed AF, compared to 144 participants in the non-MASLD group (3.14 %). In the crude model (Table 3), the RR of AF for participants with MASLD was 2.14 (95 % CI: 1.72–2.61) compared to non-MASLD participants. After adjusting for additional variables in Model 2, the RR remained similar at 2.12 (95 % CI: 1.73–2.63). In the fully adjusted Model 3, MASLD was associated with a 103 % increased risk of AF (RR = 2.03, 95 % CI: 1.67–2.47).

**Table 3:** Poisson regression between MASLD at baseline and incidence of atrial fibrillation.

		Non-MASLD	MASLD
**Atrial fibrillation**			
No. of participants		4585	3926
No. of cases		144	307
Relative Risk (95 % CI)	Model 1	Reference	2.14 (1.72–2.61)
	Model 2 Model 3	Reference Reference	2.12 (1.73–2.63)1.55 (1.12–2.13)

MASLD, metabolic dysfunction-associated steatotic liver disease; CI, confidence intervals. Model 1 was unadjusted; Model 2 was adjusted for age and sex; Model 3 was adjusted for age, sex, BMI, SBP, LDL-c, HDL-c, triglycerides, hs-CRP, GGT, estimated GFR, and all the medications.

**Fig. 2:**
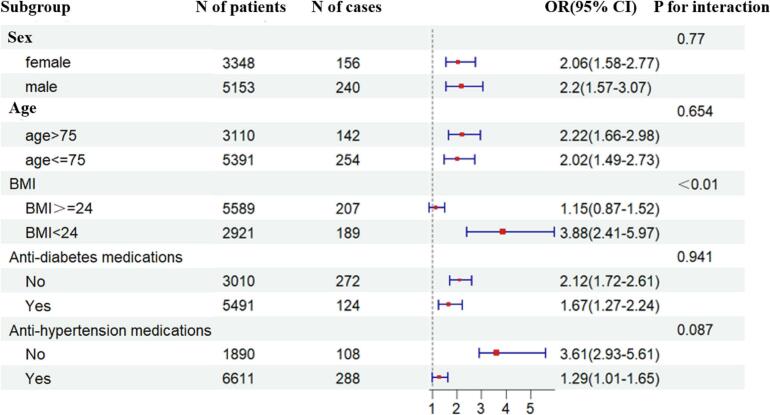
**Subgroup analysis of the association of MASLD and AF according to sex, age, BMI, anti-diabetes and anti-hypertension medications.** MASLD, metabolic dysfunction-associated steatotic liver disease; AF, atrial fibrillation; BMI, body mass index.

Subgroup analyses (Fig. 2) revealed no significant interaction between MASLD and AF based on sex (P for interaction = 0.77) or age, with a cut-off of 75 years (P for interaction = 0.654). Similarly, the use of anti-diabetes or anti-hypertensive medications did not affect the MASLD-AF association (P for interaction = 0.941 and P for interaction = 0.087, respectively). Notably, a significant interaction was observed between MASLD and BMI. The association between MASLD and AF was more pronounced in participants with a BMI < 24 kg/m^2^ compared to those with a BMI ≥ 24 kg/m^2^ (P for interaction < 0.01).

## 4 Discussion

This study highlights a significant association between MASLD and an increased risk of AF in a large cohort undergoing routine health check-ups. Even after adjusting for cardiometabolic risk factors, individuals with MASLD exhibited a higher risk of AF compared to those without MASLD. Additionally, we observed significant positive correlations between age, BMI, systolic and diastolic blood pressure, LDL levels, and AF risk. Notably, the interaction effect varied among participants with different BMI thresholds, underscoring the heterogeneity of this relationship.

Epidemiological studies have previously linked ischemic liver disease to arrhythmic events, including AF. For instance, a prospective study of 9,333 individuals without pre-existing AF reported 1,021 new cases of AF over a 12-year follow-up, with elevated liver enzymes, particularly GGT, associated with an increased risk of AF [21]. Similarly, in a Swedish cohort of adults with histologically confirmed MASLD, the incidence of arrhythmias was higher in MASLD patients (10.3 per 1,000 person-years) compared to controls (8.7 per 1,000 person-years) over a median follow-up of 10.8 years (adjusted hazard ratio [aHR] = 1.30, 95 % CI: 1.22–1.38). Specifically, MASLD patients demonstrated a significantly elevated risk of AF (aHR = 1.26, 95 % CI: 1.18–1.35). Furthermore, a *meta*-analysis by Cai *et al*. concluded that non-alcoholic fatty liver disease (NAFLD), a precursor of MASLD, is associated with a 19 % increased risk of incident AF [22]. A recent study involving a substantial cohort of anticoagulated AF patients (22,636 with NAFLD and 391,014 without liver disease) demonstrated that NAFLD was linked to a heightened risk of both thrombotic and hemorrhagic events. This emphasizes the necessity of early detection of MASLD patients at risk of AF to prevent the progression of the disease from imposing a heavier medical burden on patients [23].

Conversely, some studies have reported conflicting findings. A 2017 prospective cohort study found no direct relationship between AF and liver enzyme levels, suggesting that the onset of AF may be influenced by heart failure markers, such as N-terminal pro-brain natriuretic peptide (NT-proBNP) [24], rather than liver dysfunction. Similarly, a Mendelian randomization study proposed that NAFLD and AF are not causally related but are instead independent consequences of metabolic syndrome [25]. Long *et al*. reported that liver fat was not significantly associated with increased AF prevalence or incidence over a 12-year follow-up in a community-based longitudinal cohort [26]. Discrepancies in these findings may stem from differences in diagnostic criteria and evaluation methods for steatotic liver disease (SLD). For example, Roh *et al*. used the fatty liver index for diagnosis [27], while Labenz *et al*. relied on database records for confirmation [28]. In contrast, the present study utilized the 2023 diagnostic criteria for MASLD established by the NAFLD Nomenclature Consensus Group, which includes the presence of at least one of five cardiometabolic risk factors [29].

We identified several common risk factors for heart disease, including age, BMI, systolic blood pressure, diastolic blood pressure, LDL, and triglyceride levels, all of which were associated with an increased risk of AF. This finding suggests that the elevated risk of AF in patients with MASLD may, in part, be attributed to these co-existing factors. However, even after adjusting for multiple heart disease-related risk factors, MASLD patients exhibited a persistently elevated risk of AF, indicating that additional mechanisms may underlie the MASLD-AF association. Metabolic disturbances associated with MASLD may contribute to AF development through structural remodelling, electrical remodelling, and autonomic nervous system remodelling of the heart [30], [31]. First, interatrial septal fat thickness and left atrial stiffness have been independently linked to incident AF, potentially serving as mechanistic connections between MASLD and AF [32], [33]. Fat accumulation in the pericardium or myocardium can lead to atrial stiffness and diastolic dysfunction, both of which are significant risk factors for AF [34].

Second, as a condition characterized by metabolic inflammation, MASLD is associated with the activation of inflammatory pathways and elevated serum CRP levels [29], [35], [36]. Activation of the NLRP3 inflammasome has been linked to atrial structural changes and electrical remodelling, which may contribute to the initiation and maintenance of AF [37], [38]. Finally, insulin resistance, a hallmark of MASLD, impairs glycogen synthesis and promotes the excessive release of free fatty acids, ceramides, and liver-derived cytokines into the circulation. These metabolic disruptions can adversely affect cardiac sugar and lipid metabolism, potentially influencing AF pathogenesis [39]. While these hypotheses are biologically plausible, direct evidence is needed to confirm their roles in MASLD-related AF.

We have detected a higher risk of incident AF in BMI < 24 kg/m^2^ rather than BMI ≥ 24 kg/m^2^, probably due to the obesity paradox, which refers to the phenomenon where overweight or mildly obese patients (with higher BMI) may have better prognoses or lower risks in certain diseases compared to those with normal or lower body weight (with lower BMI) [40]. Additionally, BMI does not fully reflect overall fat distribution. Patients with a BMI < 24 may have more visceral fat, which is closely associated with metabolic dysfunction and an increased risk of cardiovascular diseases [41].

This study has several strengths. The large sample size and robust statistical analysis reduce potential errors and provide reliable results. However, some limitations should be noted. The study population was exclusively Chinese, and all participants were drawn from a single medical center, which may introduce selection bias and limit the generalizability of findings to other regions or ethnic groups. Additionally, the diagnosis of MASLD was based on imaging examinations and clinical information rather than liver biopsy, which could have provided more precise severity assessments. Similarly, AF was diagnosed using surface electrocardiogram (ECG), which does not capture the severity or staging of AF, potentially limiting the depth of analysis. Recall bias may exist due to the reliance on self-reported data and residual bias cannot be excluded since certain unmeasured or unreported risk factors—such as genetic predisposition, lifestyle variables, or medication adherence. Finally, this study is an observational study, and the chronological order of the onset of the disease may be unclear, so it is not possible to determine the causality of AF and MASLD. Additionally, there were no observed deaths during the follow-up period, which precludes performing an Aalen-Johansen competing risk analysis involving all-cause mortality.

In conclusion, participants with MASLD had a significantly higher incidence of AF compared to non-MASLD controls. These findings underscore the need for further research to validate the predictive value of early MASLD diagnosis in identifying individuals at risk for subsequent AF events.

## Funding

This work was supported by General Projects of Shanghai Municipal Health Commission (No. 202140311) and Shanghai Municipal Science and Technology Commission (No. 21ZR1477600).

## CRediT authorship contribution statement

**Yehua Tang:** Writing – original draft, Methodology, Formal analysis. **Jianling Fan:** Methodology, Formal analysis. **Xingyun Hou:** Methodology, Formal analysis. **Honghong Wu:** Writing – review & editing, Data curation. **Jiaqi Zhang:** Writing – review & editing, Data curation. **Jia Wu:** Writing – review & editing, Data curation. **Yifan Wang:** Writing – review & editing, Data curation. **Zhiyu Zhang:** Writing – review & editing, Data curation. **Bin Lu:** Writing – review & editing, Project administration, Funding acquisition. **Jiaoyang Zheng:** Writing – review & editing, Supervision, Resources, Funding acquisition.

## Declaration of competing interest

The authors declare that they have no known competing financial interests or personal relationships that could have appeared to influence the work reported in this paper.
